# ﻿Nomenclature and typification of plant names related to *Centaureaaplolepa* and *C.leucophaea* (Asteraceae) from Italy and France

**DOI:** 10.3897/phytokeys.244.127109

**Published:** 2024-07-23

**Authors:** Antonio Giacò, Lorenzo Peruzzi

**Affiliations:** 1 Plantseed Lab, Department of Biology, University of Pisa, Pisa, 56126, Italy University of Pisa Pisa Italy

**Keywords:** Endemism, epitype, ICN, lectotype, Mediterranean, neotype, taxonomy

## Abstract

*Centaureaaplolepa* Moretti and *C.leucophaea* Jord. (Asteraceae) are endemic to the central-western Mediterranean and include, respectively, ten and six subspecies, mostly occurring in north-western Italy and south-eastern France. As part of an ongoing systematic study on CentaureaL.sect.Centaurea from the central Mediterranean, 17 nomenclatural types (13 lectotypes, three neotypes and one epitype) are designated to fix the application of all names of the taxa described for France and Italy and related to *C.aplolepa* and *C.leucophaea*. In addition, previous typifications are critically revised and discussed. Centaureaaplolepasubsp.maremmana (Fiori) Dostál and *C.litigiosa* (Fiori) Arrigoni, two currently accepted taxa endemic to Tuscany (central Italy), are respectively considered here as heterotypic synonyms of C.aplolepasubsp.carueliana (Micheletti) Dostál and C.aplolepasubsp.cosana (Fiori) Dostál. Finally, C.aplolepasubsp.gallinariae (Briq. & Cavill.) Dostál, a currently accepted subspecies narrowly endemic to the Gallinara island (Liguria, northern Italy), is considered here as a heterotypic synonym of C.leucophaeasubsp.brunnescens (Briq.) Dostál.

## ﻿Introduction

*Centaurea* L. (Asteraceae), with approximately 600 currently accepted species, is one of the most species-rich genera of the Mediterranean area (Greuter 2008). Due to several biological phenomena, such as hybridization, introgression, and polyploidy, it is considered as a taxonomically critical genus. Previous phylogenetic studies, carried out by using both a nuclear (ITS) and a plastid marker (*rpl32-trnL* intergenic spacer), were able to shed light on the systematic relations among wide groups of species (Hilpold et al. 2014). However, within these groups, the relations among taxa were not resolved due to large polytomies.

Other authors, using more informative molecular approaches at finer geographic scales, were able to better clarify the taxonomy of critical species groups such as the *C.cineraria* L. group in the central Mediterranean (Hilpold et al. 2011), the *C.calocephala* Willd. group in the Balkans (Novaković et al. 2022), and the *C.tenorei* Guss. ex Lacaita group in southern Italy (De Luca et al. 2023). A great gap of taxonomic knowledge still remains for several taxa endemic to the central Mediterranean included in C.sect.Centaurea. Within this section, along with the lack of systematic studies, several accepted names (Greuter 2008), related to the two phylogenetically close species *C.aplolepa* Moretti and *C.leucophaea* Jord. (Hilpold et al. 2014), are still not typified (Peruzzi et al. 2015).

*Centaureaaplolepa* is endemic to central and north-western Italy, and its wide morphological variability is currently organized in ten subspecies (Bartolucci et al. 2024). Under the binomial *Centaureaaplolepa*, diploid (Viegi et al. 1972; Giacò et al. 2024) biennial or perennial plants growing in arid environments (limestone, sand, and ophiolites, depending on the subspecies) are included. They show glabrous to rarely tomentose pinnatisect leaves, and capitula disposed in a cymose sub-corymb; the involucral bracts show a decurrent appendage with cilia that, depending on the subspecies, can be long to very short (Arrigoni 2003). Conversely, under the binomial *C.leucophaea*, six subspecies, five of which are endemic to south-eastern France and a little portion of north-western Italy, are included. From a morphological perspective, *C.leucophaea* is similar to *C.aplolepa*, but more tomentose (Pignatti 2018).

The aim of this work is to critically revise the typifications available in literature and to typify all the remaining names (either currently accepted or synonyms) with type localities in Italy and France, which were referred in taxonomic literature to *C.aplolepa* or *C.leucophaea*. This paper is part of an ongoing integrative taxonomic study of taxa included in the section Centaurea endemic to the central Mediterranean.

## ﻿Materials and methods

Accepted names and synonyms related to *C.aplolepa* or *C.leucophaea* were searched in Greuter (2008), IPNI (2024), and WFO (2024). Protologues were investigated and original material was searched in the following herbaria: BR, CGE, G, GE, FI, LY, MPU, MW, P, PAL, PAD, PI, RO, SE, TL, and W. Some Briquet’s specimens were searched in Clarence Bicknell’s herbarium, preserved in the “Museo e Biblioteca Clarence Bicknell”, Bordighera, Imperia, Italy. Types were designated and previous typifications were critically revised following the Shenzhen Code (Turland et al. 2018, ICN hereafter). Names are listed in alphabetical order of their basionyms.

## ﻿Typifications

**1. *Centaureaaeolica* Guss. ex Lojac., Fl. Sicul. 2(1): 136. 1903 ≡ Centaureacinerariavar.aeolica (Guss. ex Lojac.) Fiori in Fiori & Paoletti, Fl. Italia 3: 334. 1904 ≡ Centaureapaniculatasubsp.aplolepavar.aeolica (Guss. ex Lojac.) Arènes in Mém. Mus. Natl. Hist. Nat., Ser. B, Bot. 1(2): 223. 1951 ≡ *Acostaaeolica* (Lojac.) Holub in Preslia 46: 226. 1974 ≡ Centaureaaplolepasubsp.aeolica (Lojac.) Dostál in Bot. J. Linn. Soc. 71: 202. 1976. Type: ITALY. Sicily: “in insula Lipari, rara et localis”, June s.d., *M. Lojacono s.n.* (lectotype, designated by Cela Renzoni and Viegi (1983: 136): PAL [barcode PAL10639] photo!, https://herbarium.unipa.it/zoomify/view_img.asp?ic=10639)**

*Centaureaaeolica* is an accepted name and applies to a species endemic to the Aeolian Islands, Sicily (Bartolucci et al. 2024).

**2. *Centaureaaplolepa* Moretti in Giorn. Fis., ser. 2, 9: 154–155. 1826 ≡ Centaureabertoloniivar.aplolepa (Moretti) Hausskn. in Mitt. Thüring. Bot. Vereins 6: 35. 1894, as “ *haplolepis* ” ≡ Centaureapaniculatavar.aplolepa (Moretti) Fiori in Fiori & Paoletti, Fl. Italia 3: 336. 1904 ≡ Centaureapaniculatasubsp.aplolepa (Moretti) Briq. & Cavill. in Burnat, Fl. Alpes Marit. 7: 172. 1931 ≡ *Acostaaplolepa* (Moretti) Holub in Preslia 45: 142. 1973. Type: ITALY. Liguria: “Caprazoppa e Promontorio di Noli”, 20 August 1824, *Moretti s.n.* (lectotype, designated here: PAD 9476! [individual and label on the right of the sheet])**

**Figure 1.**
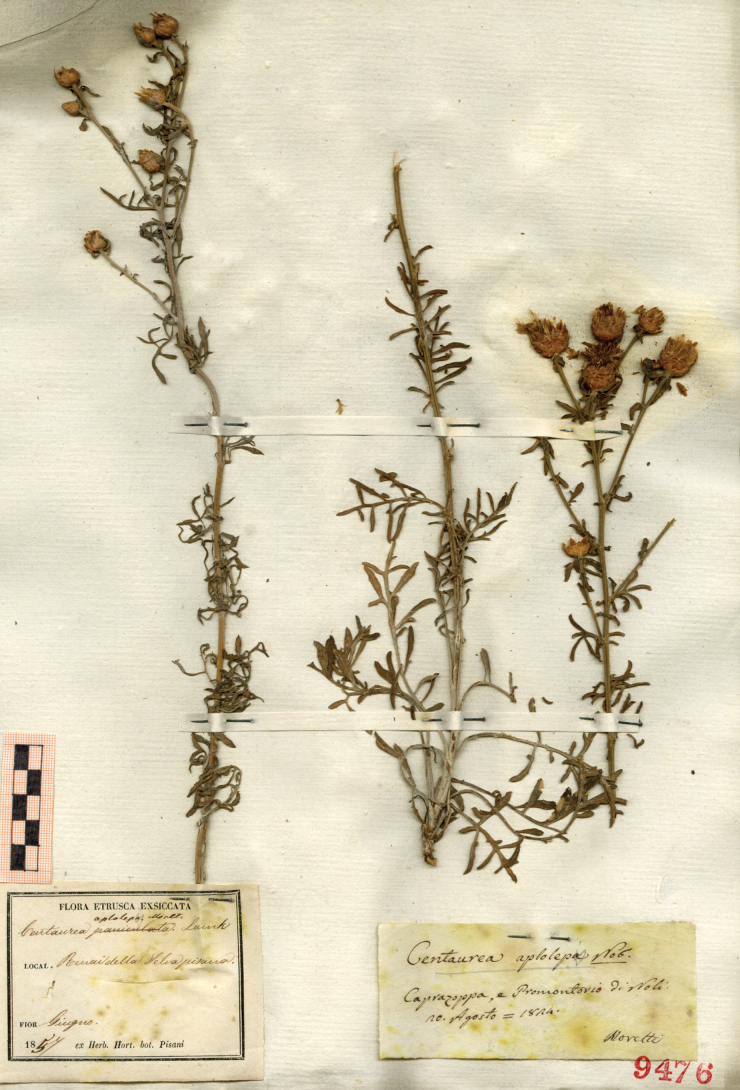
Lectotype of *Centaureaaplolepa* Moretti (individual and label on the right of the sheet). Reproduced with the permission of the herbarium of the University of Padua (PAD).

We detected a specimen (Fig. 1) at PAD collected in 1824 at “Promontorio di Noli”, in Liguria, the same date and place mentioned in the protologue (Moretti 1826). This specimen, designated as the lectotype, shows basal pedunculate pinnatisect leaves, while the cauline leaves are also pinnatisect but become progressively shorter along the branch. Capitula are globose and the involucral bracts are pointed at the apex, with few, and very short, lateral teeth. This morphology is congruent with the protologue and with the application of the name *C.aplolepa* s.str. to a taxon endemic to western Liguria (Arrigoni 2003). The name C.aplolepavar.genuina Briq. (Briquet 1902) is invalid under Art. 24.3 of the ICN.

**3. Centaureaaplolepavar.integrans Fedde, Repert. Spec. Nov. Regni Veg. 1(9): 144. 1905 ≡? Centaureapaniculataf.integrans Fiori in Fiori & Paoletti, Fl. Italia 4(1): 188. 1907 ≡? *Centaureaintegrans* Naggi ex Prain, Index Kew., Suppl. 3: 38. 1908. Type: not designated**

Naggi (1905) failed to validly publish the name *Centaureaintegrans*, since this binomial appears only in the title. Elsewhere in the protologue, in place of *C.integrans*, the author doubtfully refers the morphological description to another putative rank and epithet (“*genuensis*”), tentatively placed as a variety of *C.aplolepa*. Accordingly, the name *C.integrans* is a nomen nudum, invalid under Art. 38.1 of the ICN, and C.aplolepavar.genuensis is invalid under Art. 36.1 of the ICN. Later, Fedde (1905), Fiori (1907), and Prain (1908) independently and validly published, respectively, the names at different ranks: C.aplolepavar.integrans Fedde, C.paniculatavar.aplolepaf.integrans Fiori, and *C.integrans* Prain, in all cases referring explicitly to Naggi as the author. Accordingly, the original material for these names can be searched among those specimens belonging to Naggi, or among the specimens of each respective author (i.e., Fedde, Fiori and Prain) matching with the diagnosis provided by Naggi (1905). In the former case, the names provided by Fedde (1905), Fiori (1907), and Prain (1908) would be homotypic. Naggi (1905) described plants similar to *C.aplolepa*, but with totally entire leaves, and indicated Genoa (Liguria) as the only locality of occurrence. We searched in GE and FI, but we did not locate any pertinent *Centaurea* specimen labelled with the epithet “*integrans*” or “*genuensis*”. Similarly, we were not able to locate any pertinent specimen by Fedde, Fiori, or Prain. In the absence of original material, a neotype can be selected for each of the three validly published names. Nevertheless, based on the morphological description provided by Naggi (1905), even the designation of a neotype is not straightforward. Indeed, based on the current knowledge (Arrigoni 2003; Hilpold et al. 2011), the plants described by Naggi (1905) cannot be readily related either to *C.aplolepa* or to other similar species as *C.cineraria* L., *C.leucophaea*, or *C.paniculata* L., since they all show pinnatisect leaves. Moreover, the absence of information in Naggi (1905) concerning the morphology of involucral bracts does not allow to safely fit this description to any *Centaurea* species with entire leaves. Accordingly, we prefer to abstain from designating neotypes.

**4. Centaureaaplolepavar.ligustica Briq., Monogr. Centaurées Alpes Marit.: 142. 1902 ≡ Centaureapaniculatavar.aplolepaf.ligustica (Briq.) Fiori in Fiori & Paoletti, Fl. Italia 3: 339. 1904 ≡ *Acostaligustica* (Briq.) Holub in Preslia 46: 226. 1974 ≡ Centaureaaplolepasubsp.ligustica (Briq.) Dostál in Bot. J. Linn. Soc. 71: 202. 1976 ≡ Centaureapaniculatasubsp.ligustica (Briq.) Arrigoni in Parlatorea 6: 73. 2003. Type: ITALY. Liguria: “entre Pieve di Teco et Rezzo”, 28 July 1890, *E. Burnat and F.G. Cavillier s.n.* (lectotype, designated here: G [barcode G00848137], photo!, https://www.ville-ge.ch/musinfo/bd/cjb/chg/adetail.php?id=716970&base=img&lang=en)**

In the protologue, Briquet (1902) cited two specimens, one collected between Pieve di Teco and Rezzo (Liguria) in 1890 and another collected between Pieve di Teco and Nava (Liguria) in 1886. We located the former specimen at G, which is designated here as the lectotype. It is a tomentose plant with thin branches and small oblong capitula; the bracts at the lower portion of the involucre are dentate, whereas the ones at the upper portion show longer cilia. According to Greuter (2008), C.aplolepavar.ligustica is a heterotypic synonym of C.aplolepavar.parvula Ces. However, based on the higher degree of tomentosity and the geographical provenance of the type here designated, we deem more reliable considering it as a heterotypic synonym of C.leucophaeasubsp.brunnescens (Briq.) Dostál., a taxon endemic to northern-western Italy (Arrigoni 2003; Tison and de Foucault 2014; Pignatti 2018).

**5. Centaureaaplolepavar.parvula Ces. in Cesati & al., Comp. Fl. Ital.: 495. 1878 ≡ Centaureaaplolepasubsp.parvula (Ces.) Arcang., Comp. Fl. Ital.: 391. 1882. Type: ITALY. Piedmont: Acqui, August 1867, *V. Cesati s.n.* (lectotype, designated here: RO-HC-FAN_768, photo! [the three individuals on the left of the sheet])**

**Figure 2.**
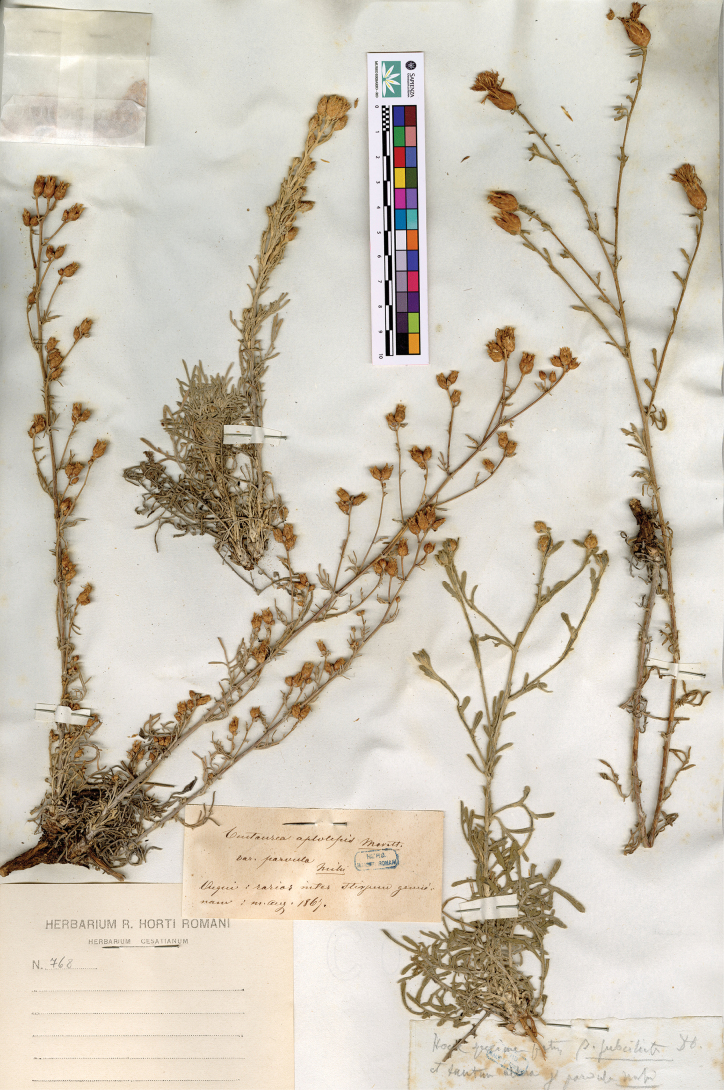
Lectotype of Centaureaaplolepavar.parvula Ces. (the three individuals on the left of the sheet). Reproduced with the permission of the herbarium RO.

We detected a specimen at RO (Fig. 2), where Cesati’s material is conserved, including four individuals. They were all collected near Acqui (Piedmont, northern Italy), the same locality mentioned in the protologue. In the label mounted at the bottom, it is reported that the individuals belong partly to C.aplolepavar.parvula Ces. and partly to C.aplolepavar.subciliata DC. Based on the label, it is not possible to attribute these plants to a single name. However, in the protologue, Cesati et al. (1878) stated that C.aplolepavar.parvula shows capitula that are two or three times smaller than those in C.aplolepavar.subciliata. With this information, it is possible to safely attribute the individual located on the right, showing larger capitula, to C.aplolepavar.subciliata sensu Cesati et al. (1878), whereas the remaining three, showing smaller capitula, can be attributed to C.aplolepavar.parvula. These three specimens are designated as the lectotype for C.aplolepavar.parvula. They are tomentose erect plants showing pinnatisect leaves; capitula are small and show involucral bracts with short teeth. This morphology is congruent with the protologue and with the application of the name C.aplolepasubsp.parvula (Ces.) Arcang. to a taxon endemic to north-western Italy (Piedmont and Liguria) (Bartolucci et al. 2024).

**6. Centaureaaplolepavar.subciliata DC., Prodr. 6: 584. 1838 ≡ Centaureaaplolepasubsp.subciliata (DC.) Arcang., Comp. Fl. Ital.: 391. 1882 ≡ Centaureapaniculatavar.aplolepaf.subciliata (DC.) Fiori in Fiori & Paoletti, Fl. Italia 3: 339. 1904 ≡ Centaureapaniculatasubsp.subciliata (DC.) Arrigoni in Parlatorea 6: 67. 2003. Type: ITALY. Tuscany: Livorno, 1832, *J.F. Schow s.n.* (lectotype, designated [as holotype] by Arrigoni (2003: 67): G [barcode G00473209], photo!, https://www.ville-ge.ch/musinfo/bd/cjb/chg/adetail.php?id=339879&base=img&lang=en)**

The name C.aplolepasubsp.subciliata (DC.) Arcang. is accepted and applies to a taxon endemic to central Italy (Tuscany) (Bartolucci et al. 2024).

**7. *Centaureabertolonii* Hausskn. in Mitt. Thüring. Bot. Vereins 6: 34. 1894, nom. illeg. (Art. 52.1) ≡ Centaureapaniculatasubsp.bertolonii Arrigoni in Parlatorea 6: 60. 2003 ≡ Centaureaaplolepasubsp.bertolonii (Arrigoni) Greuter in Willdenowia 33: 249. 2003. Type: ITALY. Liguria: Genova, in glareos vallis Bisagno, 26 August 1892, *C. Haussknecht s.n.* (lectotype, designated by Greuter (2003: 249): JE [barcode JE00010556], photo!, http://131.130.131.10/herbaria/jacq-viewer/viewer.html?rft_id=je_00010556&identifiers=je_00010556)**

The nomenclature of *Centaureabertolonii* Hausskn. was clarified by Greuter (2003). Despite the illegitimacy of this species name under Art. 52.1, due to the taxonomic inclusion of *C.aplolepa*, its typification is not automatic under Art. 7.6. The name C.aplolepasubsp.bertolonii (Arrigoni) Greuter is currently accepted and applies to plants endemic to eastern Liguria (Arrigoni 2003; Bartolucci et al. 2024).

**8. *Centaureabiformis* Timb.-Lagr. in Rev. Bot. Bull. Mens. 10: 262. 1892 ≡ Centaureapaniculatasubsp.biformis (Timb.-Lagr.) Rouy in Rev. Bot. Syst. Géogr. Bot. 2: 159. 1904 ≡ Centaureapaniculatavar.biformis (Timb.-Lagr.) Briq. & Cavill. in Burnat, Fl. Alpes Marit. 7: 191. 1931 ≡ Centaurealeucophaeasubsp.biformis (Timb.-Lagr.) Dostál in Bot. J. Linn. Soc. 71: 200. 1976. Type: FRANCE. Occitanie: “entre le Château de Caladroer et le village de Cassagnes”, 28 Juin 1881, *G. Gautier s.n.* (neotype, designated here: LY [barcode LY0000848], photo!, https://explore.recolnat.org/search/botanique/simplequery=LY0000848)**

In the protologue, Timbal-Lagrave (1892) provided a detailed morphological description and listed several localities of occurrence in Occitanie (southern France). We searched for the original material at TL, BR, CGE, FI, MPU, MW, and P, where Timbal-Lagrave’s material is known to be conserved (Stafleu and Cowan 1986), but we did not locate any specimen suitable for lectotypification. In this case, a neotype can be selected. We found several specimens (e.g. P04309069, LY0365918, LY0365919, and LY0719809) that were collected at Roquevert, near Sournia (Occitanie), one of the localities mentioned in the protologue. However, all these specimens show involucral bracts with light yellow cilia, whereas in the protologue it is stated that both forms of *C.biformis* show reddish or dark brown cilia. The specimen LY0000848 was collected between Cassagnes and Caladroer (Occitanie), at just approximately 12 km from Trevillach, one of the localities mentioned in the protologue. This specimen shows dark brown cilia and its overall morphology matches with the first of the two forms of *C.biformis* described in the protologue (Timbal-Lagrave 1892). Indeed, the plant shows a long taproot with a single stem that is branched in the upper portion. It is designated here as the neotype for *C.biformis*. Centaurealeucophaeasubsp.biformis (Timb.-Lagr.) Dostál is a name accepted by Greuter (2008), albeit Tison and de Foucault (2014) considered *C.biformis* as a heterotypic synonym of *C.leucophaea* s.str. Timbal-Lagrave (1892) suggested that *C.biformis* is included in the group of *C.maculosa* Lam. (= *C.stoebe* L.). Based on the morphology of the neotype here designated, we confirm the observations made by the latter author, so that *C.biformis* has to be considered a heterotypic synonym of *C.stoebe*, a species widespread in central-eastern Europe (Greuter 2008).

**9. Centaureacinerariavar.pandataria Fiori & Bég. in Fiori & Paoletti, Fl. Italia 3: 334. 1904 ≡ *Centaureapandataria* (Fiori & Bég.) Bég. in Ann. Bot. (Rome) 3: 443. 1905 ≡ Centaureaaplolepasubsp.pandataria (Fiori & Bég.) Dostál in Bot. J. Linn. Soc. 71: 202. 1976 ≡ Centaureaaeolicasubsp.pandataria (Fiori & Bég.) Anzal. in Boll. Soc. Sarda Sci. Nat. 30: 512. 1995. Type: ITALY. Lazio: sulle rupi maritime a Ventotene, 20 September 1901, *A. Béguinot s.n.* (lectotype designated by Brullo et al. (2021: 17): FI [barcode FI051939!])**

This taxon was considered as a subspecies of *C.aeolica* by Greuter (2008). After Brullo et al. (2021) and Del Guacchio et al. (2022), it is considered as a distinct species, endemic to Ventotene island (Lazio).

**10. *Centaurealeucophaea* Jord., Observ. Pl. Nouv. 5: 64. 1847 ≡ Centaureapaniculatasubsp.leucophaea (Jord.) Arcang., Comp. Fl. Ital.: 392. 1882 ≡ Centaureapaniculatavar.leucophaea (Jord.) Briq., Monogr. Centaurées Alpes Marit.: 147. 1902 ≡ *Acostaleucophaea* (Jord.) Holub in Folia Geobot. Phytotax. 7: 314. 1972. Type: [Illustration] “F. *C. leucophæa* ” in Jordan, Observ. Pl. Nouv. 5: Pl 4. 1847 (lectotype, designated here). FRANCE. Provence-Alpes-Côte d’Azur: De Brumebou, près Serres, H. Alpes, 3 July 1847, *A. Jordan s.n.* (epitype, designated here: LY [barcode LY0799389], photo!, https://explore.recolnat.org/occurrence/D6453FD94C5A46BBBC365B9DBC1D970F ; isoepitype: LY [barcode LY0799390], photo!)**

In the protologue, Jordan (1847) reported a detailed description and several diagnoses; he also mentioned several localities in Provence-Alpes-Côte d’Azur (southern France): Briançon, Guillestre, Gap, Serres, Sisteron, and Castellane. We found two specimens at LY (LY0799389 and LY0799390) that were collected near Serres in July 1847 (interpretation of the calligraphy confirmed by the LY curator M. Thiébaut, pers. comm.). However, these two specimens are not original material since the name was published six months earlier (Stafleu and Cowan 1979). Along with the protologue, Jordan (1847) provided an illustration in which a capitulum, an involucral bract, and a cypsela of *C.leucophaea* are depicted. The illustration is the only available original material and is designated as the lectotype. However, such a minimalistic illustration does not provide an exhaustive interpretation of the overall plant morphology. Indeed, several characters, mostly related to the leaf morphology, mentioned in the protologue as discriminant with respect to other species, are missing in the illustration. For this reason, since the lectotype is ambiguous and does not allow a clear identification of the taxon, following Art. 9.9 of the ICN, we designate the above mentioned specimen LY0799389 as the epitype for *C.leucophaea*. The specimen shows capitula and involucral bracts as in the lectotype illustration, and pinnatisect tomentose leaves with oblong lobes. This morphology is congruent with the protologue and with the application of the name *Centaurealeucophaea* to a species native to Spain, France, and Italy (Greuter 2008). According to the latter author, this species includes six subspecies, and our typification confirms the application of *C.leucophaea* s.str. to plants endemic to southern France and north-western Italy, as also circumscribed by Arrigoni (2003). The name C.paniculatavar.euleucophaea Briq. (Monogr. Centaurées Alpes Marit.: 149. 1902) is invalid under Art. 24.3 of the ICN.

**11. *Centaureamierghii* Jord., Cat. Graines Jard. Bot. Grenoble: 15. 1850 ≡ *Acrolophusmierghii* (Jord.) Fourr. in Ann. Soc. Linn. Lyon, sér. 2, 17: 97. 1869 ≡ Centaureapaniculatasubsp.leucophaeavar.mierghii (Jord.) Rouy in Rev. Bot. Syst. Geogr. Bot. 2: 148. 1904. Type: FRANCE. Provence-Alpes-Côte d’Azur: Lyon [plants cultivated from seeds collected in Occitanie, Anduze], 24 June 1851, *A. Jordan s.n.* (neotype, designated here: LY [barcode LY0368554], photo!, https://explore.recolnat.org/occurrence/E92AB6A81F9147258A3B77D327171727)**

In the protologue, Jordan (1850) stated that he had grown plants in his garden from *Centaurea* seeds sampled by Miergue in Occitanie. At LY, we found two specimens (LY0368554 and LY0368558) that were collected in Jordan’s garden (“mj” = mon jardin [my garden]) in 1851 and 1852, respectively. On the same labels, it is reported “Anduze, Gard 1849”, so it is plausible to assume that 1849 is the date of collection of seeds in Anduze (Occitanie), one of the localities mentioned in the protologue. It is plausible, that the protologue was based on living plants grown in Jordan’s garden from seeds collected in Anduze, later herborized in 1851 and 1852 (M. Thiébaut, pers. comm.). However, following Art. 9.4 of the ICN, since both specimens were collected after the publication of the name, they cannot be considered as original material. No illustration is provided in the protologue, so that following Art. 9.8 of the ICN a neotype can be selected. Both specimens show ovoid-oblong involucres with brown appendages and lateral upper cilia that exceed in height the central mucro. Cauline leaves are pinnatisect and are composed by several linear segments. This morphology is in accordance with the protologue, and the above-mentioned specimen LY0368554 is designated as the neotype for *C.mierghii*. Based on the neotype morphology, we agree with Greuter (2008) in considering *C.mierghii* as a heterotypic synonym of *C.stoebe*, a species widespread in central and eastern Europe.

**12. Centaureapaniculatavar.aetaliae Sommier in Nuovo Giorn. Bot. Ital. 9: 329. 1902 ≡ *Centaureaaetaliae* (Sommier) Bég. in Arch. Bot. (Forlì) 7: 93. 1931 ≡ Centaureaaplolepasubsp.aetaliae (Sommier) Dostál in Bot. J. Linn. Soc. 71: 203. 1976. Type: ITALY. Tuscany: Insula Elba, valle di Monserrato in rupibus, 17 June 1900, *S. Sommier s.n.* (lectotype, designated by Arrigoni (2020: 372): FI [barcode FI002032!])**

*Centaureaaetaliae* is an accepted name and applies to a species endemic to the eastern portion of Elba island, Tuscany (Arrigoni 2003, Bartolucci et al. 2024).

**13. Centaureapaniculatavar.aetaliaef.maremmana Fiori in Fiori & Paoletti, Fl. Italia 3: 339. 1904 ≡ Centaureapaniculatavar.maremmana (Fiori) Fiori, Nuov. Fl. Italia 2: 732. 1927 ≡ Centaureaaplolepasubsp.maremmana (Fiori) Dostál in Bot. J. Linn. Soc. 71: 202. 1976 ≡ Centaureapaniculatasubsp.maremmana (Fiori) Arrigoni in Parlatorea 6: 71. 2003. Type: ITALY. Tuscany: presso M. Cerboli, s.d., *Amidei s.n.* (lectotype, designated by Arrigoni (2003: 71): FI!)**

Fiori (1904) provided a short diagnosis for C.paniculataf.maremmana and cited two collections from Tuscany (“*M. Cerboli*, Amidei in hb. flor.!” and “*Castiglioncello*, Campana in hb. flor.!”) conserved at FI. The specimen from Montecerboli was designated as the lectotype by Arrigoni (2003). Later, the same author (Arrigoni 2012) revised his previous typification by stating that the lectotype is in conflict with the original description for showing shortly ciliate, and not dentate, involucral bracts. Accordingly, the other specimen from Castiglioncello, showing dentate involucral bracts, was designated as a new lectotype for C.paniculataf.maremmana. Indeed, the Melbourne Code (McNeill et al. 2012) allowed to resolve these cases through Art. 9.19(b), albeit this article was not mentioned by Arrigoni (2012). However, the current Code (Turland et al. 2018), due to Note 7, does not allow to apply Art. 9.19(c) when gatherings are explicitly cited in the protologue (i.e. syntypes), as in this case. Thus, the second typification provided by Arrigoni (2012) is not effective and the first lectotypification made, despite being perhaps less accurate, is final. The lectotype is glabrous and shows pinnatisect leaves with linear lobes; its capitula are small (diameter 4–5 mm, and the involucral bracts are shortly ciliate. Based on this lectotype, as also stated by Arrigoni (2012), C.paniculataf.maremmana has to be considered a heterotypic synonym of C.aplolepasubsp.carueliana (Micheletti) Dostál, a taxon endemic to Tuscany, central Italy. More studies are needed to understand if the populations growing in the areas surrounding Castiglioncello, showing dentate involucral bracts, deserve a distinct taxonomic treatment with respect to the typical population, showing shortly dentate involucral bracts.

**14. Centaureapaniculatavar.aplolepaf.virescens Fiori in Fiori & Paoletti, Fl. Italia 3: 339. 1904. Type: ITALY. Tuscany: “pineta di Viareggio”, 10 September 1903, *A. Fiori s.n.* (lectotype, designated here: FI! [the individual on the upper portion of the sheet])**

Fiori (1904) described C.paniculatavar.aplolepaf.virescens as “quasi glabra e verde” [almost glabrous and green] comparing it to the white tomentose C.paniculatavar.aplolepaf.subciliata (DC.) Fiori. Contrary to the usual procedure, the author did not provide a Latin letter for C.paniculataf.virescens, or mention localities of occurrence or herbarium specimens. We searched in FI, where Fiori’s herbarium is conserved, but we did not locate any specimen signed by him as “*virescens*”. Since the intention of the author was to describe individuals of C.aplolepasubsp.subciliata showing less tomentosity, we deem that the original material of C.aplolepaf.virescens has to be searched among those specimens identified by Fiori as C.aplolepasubsp.subciliata and showing less tomentum. We located a single specimen matching these features that was collected before of the publication of the name. This herbarium sheet is composed by a complete individual showing just sparse tomentum, and by two further distinct tomentose basal rosettes. The former is designated as the lectotype for C.aplolepaf.virescens. Since the variability of C.aplolepasubsp.subciliata includes white tomentose to almost glabrous plants (Arrigoni 2003), C.aplolepaf.virescens is just a heterotypic synonym of C.aplolepasubsp.subciliata.

**15. Centaureapaniculatavar.brunnescens Briq., Monogr. Centaurées Alpes Marit.: 152. 1902 ≡ Centaureapaniculatavar.leucophaeaf.brunnescens (Briq.) Fiori in Fiori & Paoletti, Fl. Italia 3: 337. 1904 ≡ Centaurealeucophaeasubsp.brunnescens (Briq.) Dostál in Bot. J. Linn. Soc. 71: 200. 1976 ≡ Centaureapaniculatasubsp.brunnescens (Briq.) Arrigoni in Parlatorea 6: 55. 2003. Type: ITALY. Liguria: “lungo la strada fra Pigna e Monte Cavanelli”, 13 October 1893, *C. Bicknell s.n.* (lectotype, designated here: Herbarium Bicknell, 23b-30-1 photo!, conserved in the herbarium of the Museo e Biblioteca Clarence Bicknell, Bordighera, Imperia, Italy)**

**Figure 3.**
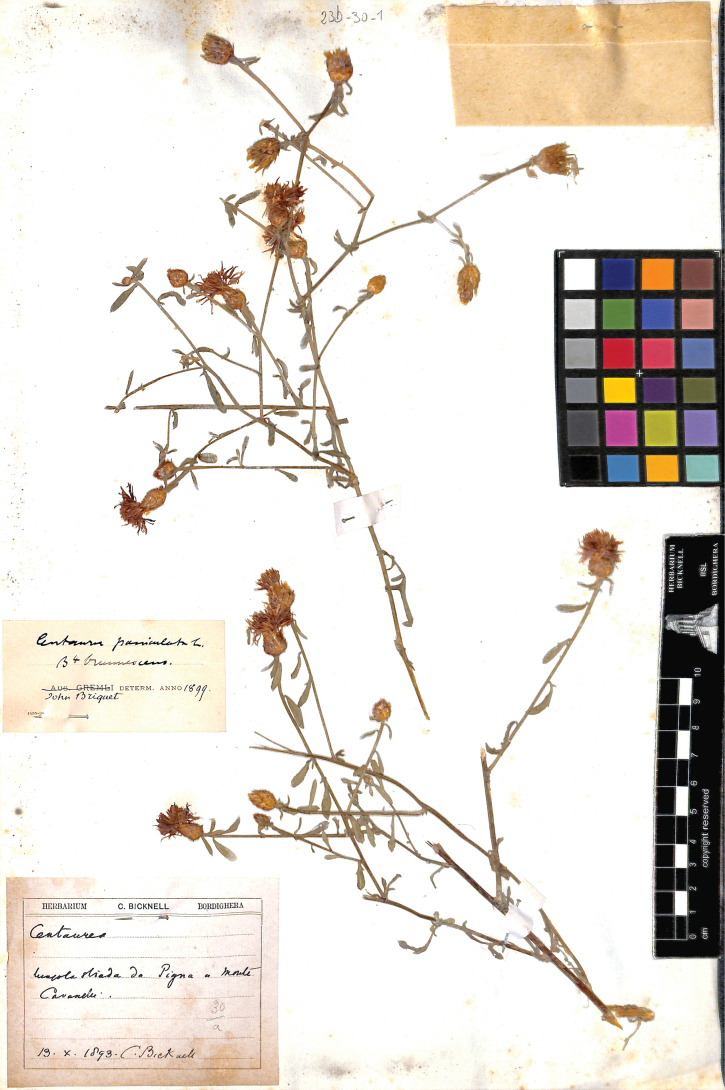
Lectotype of Centaureapaniculatavar.brunnescens Briq. Reproduced with the permission of the herbarium of the Museo e Biblioteca Clarence Bicknell, Bordighera, Liguria, Italy.

In the protologue, Briquet (1902) mentioned eight specimens from the following localities: Albenga (Savona, Liguria), Gallinara island (Savona, Liguria), Oneglia valley (Imperia, Liguria), between San Bartolomeo and San Bernardo (Imperia, Liguria), Porto Maurizio (Imperia, Liguria), between Pigna and Monte Cavanelle (Imperia, Liguria), Roquebrune (Alpes Maritimes, Provence-Alpes-Côte d’Azur), and Peïra Cava (Alpes Maritimes, Provence-Alpes-Côte d’Azur). Peruzzi et al. (2015) suggested that the lectotype has to be selected among the specimens collected “between San Bartolomeo and San Bernardo” or “between Pigna and Monte Cavanelle”, since the six remaining localities fall in the circumscription of other currently accepted taxa as C.paniculatasubsp.paniculata, C.leucophaeasubsp.leucophaea, and C.aplolepasubsp.gallinariae. In the Herbarium Bicknell, we located a specimen revised by Briquet that was collected between Pigna and Monte Cavanelle, that is designated here as the lectotype (Fig. 3). It is composed of two tomentose flowering branches showing capitula with ovoid involucres (1–1.2 × 0.8–1 mm) and large involucral bracts; the appendages are fawn and cilia are around 1 mm long. This morphology is congruent with the protologue and with the application of the name C.leucophaeasubsp.brunnescens to plants endemic to western Liguria (Arrigoni 2003; Tison and de Foucault 2014; Bartolucci et al. 2024).

**16. Centaureapaniculataf.carueliana Micheletti in Nuovo Giorn. Bot. Ital. 23: 316. 1891 ≡ Centaureapaniculatavar.carueliana (Micheletti) Fiori in Fiori & Paoletti, Fl. Italia 3: 335. 1904 ≡ Centaureaaplolepasubsp.carueliana (Micheletti) Dostál in Bot. J. Linn. Soc. 71: 202. 1976 ≡ Centaureapaniculatasubsp.carueliana (Micheletti) Arrigoni in Parlatorea 6: 69. 2003. Type: ITALY. Tuscany: Monte Ferrato (Agro fiorentino), 27 July 1857, *Pirotta s.n.* (lectotype designated by Arrigoni (2003: 69): FI!)**

Centaureaaplolepasubsp.carueliana (Micheletti) Dostál is an accepted name that applies to plants endemic to Tuscany, central Italy (Arrigoni 2003; Bartolucci et al. 2024).

**17. Centaureapaniculatasubsp.controversa Briq. & Cavill. in Burnat, Fl. Alpes Marit. 7: 182. 1931 ≡ Centaurealeucophaeasubsp.controversa (Briq. & Cavill.) Kerguélen in Lejeunia, ser. 2, 120: 65. 1987. Type: FRANCE. Provence-Alpes-Côte d’Azur: Baus-Rous, près Villefranche, 20 June 1872, *C. Sarato s.n.* (lectotype, designated here: G [barcode G00848147], photo!, https://www.ville-ge.ch/musinfo/bd/cjb/chg/adetail.php?id=728535&lang=en)**

In the protologue, Briquet and Cavillier (1931) cited several specimens collected at Baus Rous, Provence-Alpes-Côte d’Azur, southern France. We located at G the specimen collected by C. Sarato in 1872 near Villefranche, at Baus Rous. It is a white tomentose plant with capitula 8–12 mm large showing dark appendages and long cilia. As stated in the protologue, the main diagnostic character with respect to C.leucophaeasubsp.leucophaea lie in the involucral bracts, which show prominent veins. According to Greuter (2008), C.leucophaeasubsp.controversa (Briq. & Cavill.) Kerguélen is an accepted name that applies to plants endemic to southern France. However, based on the lectotype, we agree with Tison and de Foucault (2014) in considering this name as a heterotypic synonym of *C.pseudocineraria* (Fiori) Rouy, a species endemic to Provence-Alpes-Côte d’Azur.

**18. Centaureapaniculatavar.cosana Fiori in Fiori & Paoletti, Fl. Italia 3: 338. 1904 ≡ Centaureaaplolepasubsp.cosana (Fiori) Dostál in Bot. J. Linn. Soc. 71: 203. 1976 ≡ Centaureapaniculatasubsp.cosana (Fiori) Arrigoni in Parlatorea 6: 73. 2003. Type: ITALY. Tuscany: Monte Argentario verso Porto Ercole, 8 July 1886, *A. Fiori s.n.* (lectotype, designated by Baldini (1995: 147): FI [barcode FI002013]!)**

Centaureaaplolepasubsp.cosana (Fiori) Dostál is an accepted name that applies to plants endemic to southern Tuscany, central Italy (Arrigoni 2003; Bartolucci et al. 2024).

**19. Centaureapaniculatavar.cosanaf.litigiosa Fiori in Fiori & Paoletti, Fl. Italia 3: 338. 1904 ≡ Centaureapaniculatavar.litigiosa (Fiori) Sommier in Nuovo Giorn. Bot. Ital. n.s. 19: 121. 1912 ≡ Centaureapaniculatasubsp.aplolepavar.cosanasubvar.litigiosa (Fiori) Arènes in Mém. Mus. Natl. Hist. Nat., Ser. B, Bot. 1: 226. 1951 ≡ *Centaurealitigiosa* (Fiori) Arrigoni in Parlatorea 6: 77. 2003. Type: ITALY. Tuscany: sopra Port’Ercole, Monte Argentario, 5 July 1873, *H. Groves s.n.* (lectotype, designated by Baldini (1995: 147): FI [barcode FI002008!]; isolectotypes: FI [barcode FI002062!], FI [barcode FI002063!])**

In the protologue of C.paniculatavar.cosana, Fiori (1904) described also C.paniculatavar.cosanaf.litigiosa providing the following diagnosis: “a rami brevi, in pianta ricordante la *C.dissecta* v. *ilvensis* (cioè a capolini un po’ più grandi, meno strozzati all’apice ed a ciglia delle squame più lunghe)” [branches short, plants reminding C.dissectavar.ilvensis (i.e. with capitula slightly larger, less tight at the apex, and involucral bracts with longer cilia)]. Both C.paniculatavar.cosana and C.paniculatavar.cosanaf.litigiosa were described based on material from Porto Ercole, on the promontory of Monte Argentario (Tuscany, central Italy). The original material of C.paniculataf.litigiosa, conserved at FI, consists of four individuals of the same gathering mounted on two distinct herbarium sheets. On the first sheet, the individual under barcode FI002062 is mounted, whereas on the second sheet, the three remaining individuals (one under barcode FI002008 and two under barcode FI002063) can be found. Baldini (1995) designated the individual under barcode FI002008 as the lectotype. It is a small but fully developed plant showing branches shorter than the lectotype of C.paniculatavar.cosana. This morphology is in accordance with the protologue and the choice made by Baldini (1995) is accurate, since the individual under the barcode FI002062 shows branches that are even longer than the lectotype of C.paniculatavar.cosana. Sommier (1912), based on morphological observations conducted on further material collected by himself on the promontory of Monte Argentario, proposed the combination C.paniculatavar.litigiosa. According to this author, C.paniculatavar.litigiosa, if compared to C.paniculatavar.cosana, shows short, entirely prostrate, and densely foliose branches, ovate capitula, and involucral bracts with dark appendages. In the same publication, Sommier (1912) pointed out the presence of individuals with a suberect habitus and a general morphology that is intermediate between C.paniculatavar.cosana and C.paniculatavar.litigiosa. These intermediates were interpreted as a new taxon, namely C.paniculatavar.litigiosaf.suberecta Sommier. Later, Arrigoni (2003), possibly based on the observations made by Sommier (1912), raised C.paniculataf.litigiosa at species rank, then followed by Greuter (2008) and Bartolucci et al. (2024). Arrigoni (2020) interpreted the individuals showing an intermediate morphology as hybrids between C.paniculatavar.cosana and *C.litigiosa*. However, some of the characters used by Sommier (1912) and Arrigoni (2003, 2020) to circumscribe C.paniculataf.litigiosa, such as the entirely prostrate branches and the dark appendages, are neither shown by the lectotype of C.paniculataf.litigiosa, nor by the isolectotypes. These features can be observed only in some of the specimens from Monte Argentario collected later by Sommier. Accordingly, the current taxonomic position of *C.litigiosa* (Fiori) Arrigoni as a species distinct from C.aplolepasubsp.cosana is based on a misinterpretation of the former taxon. After critically checking and comparing the original material and types of both taxa, our conclusion is that C.paniculataf.litigiosa can be considered just as a heterotypic synonym of C.paniculatavar.cosana. In this perspective, the latter taxon, currently accepted as C.aplolepasubsp.cosana, includes individuals with both short and long erect and suberect branches, capitula with involucres 6–8 mm large, and light yellow to brown appendages with cilia at most 0.8 mm long. More studies are needed to understand whether the individuals showing only prostrate branches, larger involucres with dark brown to black appendages and longer cilia, ascribed by Sommier (1912) to C.paniculataf.litigiosa, are actually a distinct taxon or are part of the variability of C.aplolepasubsp.cosana. The occurrence of individuals showing an intermediate morphology seemingly supports the latter option. This latter interpretation is supported also by Baldini (1995), who observed that C.paniculatavar.cosana occurs in ruderal and disturbed areas, whereas C.paniculataf.litigiosa sensu Sommier (1912) grows on cliffs. Then, the distinct morphology could be just a consequence of local adaptations or morphological plasticity induced by the environment.

**20. Centaureapaniculatavar.gallinariae Briq. & Cavill. in Burnat, Fl. Alpes Marit. 7: 175. 1931 ≡ Centaureaaplolepasubsp.gallinariae (Briq. & Cavill.) Dostál in Bot. J. Linn. Soc. 71: 203. 1976 ≡ Centaureapaniculatasubsp.gallinariae (Briq. & Cavill.) Arrigoni in Parlatorea 6: 55. 2003. Type: ITALY. Liguria: “Ile de Gallinara”, 2 July 1880, *E. Burnat s.n.* (lectotype, designated here: G [barcode G00848144], photo!, https://www.ville-ge.ch/musinfo/bd/cjb/chg/adetail.php?id=716981&base=img&lang=en)**

Briquet and Cavillier (1931) described this taxon based on material from the Gallinara island, western Liguria, northern Italy. We located a specimen at G, identified by Briquet as C.paniculatavar.gallinariae, that was collected by E. Burnat in 1880 at Gallinara island. This specimen is a tomentose plant with pinnatisect leaves composed of several linear lobes. Capitula are isolated or grouped by two or three and are shortly pedunculate; the involucre is ovoid and is composed by ciliate bracts. This morphology is congruent with the protologue and the specimen is designated here as the lectotype. Briquet (1902) formerly included this taxon within the variability of C.paniculatavar.brunnescens. When describing C.paniculatavar.gallinariae, Briquet and Cavillier (1931) stated that the only discriminant character between these two taxa lies in the morphology of the lower involucral bracts, which show entire to sub-entire, and just rarely scarcely dentate, margin in C.paniculatavar.gallinariae. Currently, this taxon is accepted as C.aplolepasubsp.gallinariae and applies to plants narrowly endemic to the Gallinara island, Liguria, northern Italy (Greuter 2008, Bartolucci et al. 2024). However, the only discriminant character mentioned by Briquet and Cavillier (1931) has no taxonomic value, since also the type of C.paniculatavar.brunnescens shows lower bracts with sub-entire to rarely dentate margins. Thus, C.paniculatavar.gallinariae should be considered as a heterotypic synonym of C.leucophaeasubsp.brunnescens.

**21. Centaureapaniculatasubsp.levantina Arrigoni in Parlatorea 6: 62. 2003 ≡ Centaureaaplolepasubsp.levantina (Arrigoni) Greuter in Willdenowia 33: 249. 2003. Type: ITALY. Liguria: “Rupi marittime tra Sestri Levante e Lavagna”, 4 July 1977, *P.V. Arrigoni & al. s.n.* (holotype: FI [barcode FI002314]!)**

The name C.aplolepasubsp.levantina (Arrigoni) Greuter is accepted and applies to plants endemic to eastern Liguria (Arrigoni 2003; Bartolucci et al. 2024).

**22. Centaureapaniculatavar.lunensis Fiori in Fiori & Paoletti, Fl. Italia 3: 338. 1904 ≡ Centaureaaplolepaf.lunensis (Fiori) Gugler, Centaur. Ungar. (Ann. Mus. Nat. Hungar. VI.): 162. 1907 ≡ Centaureaaplolepasubsp.lunensis (Fiori) Dostál in Bot. J. Linn. Soc. 71: 203. 1976 ≡ Centaureapaniculatasubsp.lunensis (Fiori) Arrigoni in Parlatorea 6: 64. 2003. Type: ITALY. Liguria: “Bocca di Magra”, July 1873, *H. Groves s.n.* (lectotype, designated by Arrigoni (2003: 64): FI [barcode FI002012!])**

The name C.aplolepasubsp.lunensis (Fiori) Dostál is accepted and applies to plants endemic to eastern Liguria and small portions of Emilia-Romagna and Tuscany, central and northern Italy (Arrigoni 2003; Bartolucci et al. 2024).

**23. Centaureapaniculatasubsp.pallidula Rouy in Rev. Bot. Syst. Geogr. Bot. 2: 147. 1904 ≡ Centaureapaniculatasubsp.leucophaeasubvar.pallidula (Rouy) Arènes in Mém. Mus. Natl. Hist. Nat., Ser. B, Bot. 1(2): 203. 1951. Type: FRANCE. Occitanie: Cerdagne, 1 or 5 August 1902, *F. Sennen s.n.* (lectotype, designated here: LY [barcode LY0000818], photo! (the individual at the upper portion of the sheet, on the left), https://explore.recolnat.org/occurrence/DF9CA0CBBEAB4A8C9A4CE4407420D52D)**

In the protologue (Rouy 1904) mentioned a gathering collected by himself and F. Sennen at Cerdagne (Pyrénées-Orientales, Occitanie). The specimen LY0000818, conserved in Rouy’s herbarium, matches with the information provided in the protologue. On the herbarium sheet, five individuals, collected on the 1^st^ and 5^th^ August 1902, are mounted. The morphology of all the individuals agrees with the protologue, since they show the general morphology of *C.leucophaea* but with light green leaves and involucral bracts with pale fawn appendices and whitish cilia. In addition, we remark the presence, not mentioned in the protologue, of involucral bracts with a peculiar membranous appendices connecting the lower cilia. The best preserved and most developed individual, mounted at the upper portion of the sheet on the left, is designated as the lectotype. Since it is not possible to know the precise date of collection, the remaining four individuals cannot be safely considered as isolectotypes, since they can possibly belong to a distinct gathering.

Greuter (2008) considered this taxon as a heterotypic synonym of C.paniculatasubsp.paniculata. However, based on the peculiar lectotype morphology, we agree with Tison and de Foucault (2014) considering C.paniculatasubsp.pallidula as a distinct taxon endemic to Pyrénées-Orientales, southern France.

**24. Centaureapaniculatavar.pseudocineraria Fiori in Fiori & Paoletti, Fl. Italia 3: 338. 1904 ≡ *Centaureapseudocineraria* (Fiori) Rouy in Rev. Bot. Syst. Géogr. Bot. 2: 141. 1904 ≡ Centaureapaniculatasubsp.pseudocineraria (Fiori) Arènes in Mém. Mus. Natl. Hist. Nat., Ser. B, Bot. 1(2): 206. 1951. Type: FRANCE. Provence-Alpes-Côte d’Azur: Baus-Rous, between Beaulieu and Èze, 21 May 1889, *H. Groves s.n.* (lectotype, designated here: FI!)**

In the protologue, Fiori (1904) provided a short diagnosis and reported Villafranca (Baus Rous, Provence-Alpes-Côte d’Azur, France) as locality of occurrence. We located two specimens in FI that were identified by Fiori as C.paniculatavar.pseudocineraria. They were both collected before the publication of the name near Villafranca, the place mentioned in the protologue. We selected the best preserved specimen, collected by Groves in 1889, as the lectotype. It is a white tomentose plant, 30 cm tall, with capitula 12 mm large showing a dark appendage and long cilia. This morphology is congruent with the protologue and with the application of the name *C.pseudocineraria* (Fiori) Rouy to a species endemic to Provence-Alpes-Côte d’Azur (Tison and de Foucault 2014).

**25. Centaureapaniculatavar.pseudocoerulescens Briq., Cent. Alp. Marit.: 148. 1902 ≡ Centaureapaniculatavar.leucophaeaf.pseudocoerulescens (Briq.) Fiori in Fiori & Paoletti, Fl. Italia 3: 337. 1904 ≡ Centaurealeucophaeasubsp.pseudocoerulescens (Briq.) Dostál in Bot. J. Linn. Soc. 71: 200. 1976. Type: FRANCE. Provence-Alpes-Côte d’Azur: “près d’Aurent (environs d’Annot)”, 21 July 1885, *E. Burnat s.n.* (lectotype, designated here: G [barcode G00628035], photo!; isolectotype: G [barcode G00628034], photo!)**

In the protologue, Briquet (1902) provided a short diagnosis and cited several herbarium specimens. We located five of them at G and the best preserved and complete specimen (G00628035) is designated as the lectotype. It is a white tomentose plant, with large ovoid involucres, and involucral bracts with dark brown appendages and long cilia. Based on this morphology, we agree with Greuter (2008) and Tison and de Foucault (2014) considering C.paniculatavar.pseudocoerulescens as a heterotypic synonym of *C.pseudocineraria* (Fiori) Rouy, a species endemic to Provence-Alpes-Côte d’Azur, southern France.

**26. Centaureapaniculatavar.valesiaca DC., Prodr. 6: 584. 1838 ≡ *Centaureavalesiaca* (DC.) Jord. in Mém. Acad. Roy. Sci. Lyon, Sect. Lett., ser. 2 1: 322. 1851 ≡ Centaureapaniculatasubsp.valesiaca (DC.) Nyman, Consp. Fl. Eur.: 426. 1879 ≡ Centaureamaculosavar.valesiaca (DC.) Gugler in Centaur. Ungar. (Ann. Mus. Nat. Hungar. VI.): 167. 1907 ≡ *Acostavalesiaca* (DC.) Holub in Preslia 46: 227. 1974. Type: SWITZERLAND. Valais: Simplon, 1824, *M.N. Puerari s.n.* (lectotype, designated here: G [barcode G00473215] photo!, https://www.ville-ge.ch/musinfo/bd/cjb/chg/adetail.php?id=339867&base=img&lang=en)**

In the protologue, Candolle (1838) provided a short diagnosis and indicated the canton of Valais (Switzerland) as region of occurrence. We located a specimen (G00473215) in Candolle’s herbarium that is original material. It was collected in 1824 in Simplon (Valais, Switzerland), in the geographic circumscription mentioned by Candolle (1838). The specimen is in accordance with the short morphological description reported in the protologue (almost hairless leaves, pinnatisect with acute segments), and is designated as the lectotype. This taxon was formally placed under *C.leucophaea* by Rouy (1904: 148) as C.paniculatasubsp.leucophaeavar.valesiaca. According to Greuter (2008), *C.valesiaca* (DC.) Jord. is accepted and applies to a species native to Switzerland, France and western-northern Italy. Later, Tison and de Foucault (2014) excluded France from the range of this species, while Bartolucci et al. (2024) confirmed its occurrence in Italy.

**27. *Centaureareuteri* Rchb.f. in Reichenbach, Icon. Fl. Germ. Helv. 15: 33. 1852 ≡ Centaureapaniculatasubsp.reuteri (Rchb.f.) Nyman, Consp. Fl. Eur.: 426. 1879 ≡ Centaureapaniculatavar.reuteri (Rchb.f.) Briq., Cent. Alp. Marit.: 151. 1902 ≡ Centaureapaniculataf.reuteri (Rchb.f.) Fiori in Fiori & Paoletti, Fl. Italia 3: 337. 1904 ≡ Centaurealeucophaeavar.reuteri (Rchb.f.) Gugler, Centaur. Ungar. (Ann. Mus. Nat. Hungar. VI.): 177. 1907 ≡ Centaurealeucophaeasubsp.reuteri (Rchb.f.) Dostál in Bot. J. Linn. Soc. 71: 200. 1976. Type: [Illustration] t. 49 I (1–9), in Reichenbach, Icon. Fl. Germ. Helv. 15. 1852 (lectotype, designated here)**

In the protologue, Reichenbach (1852) cited two specimens: one collected by himself and G.F. Reuter at Col de Braus (Provence-Alpes-Côte d’Azur), close to the current Italian border, and another specimen collected by J.P. Barla at Cimiez (Nice, Provence-Alpes-Côte d’Azur). We searched for original material at W, where H.G. Reichenbach’s herbarium is conserved (Stafleu and Cowan 1983), but we did not locate any specimen. Along with the protologue, the illustration (t. 49 I, 1–9) of a green plant with pinnatisect leaves and ovoid capitula with involucral bracts showing fawn and long cilia is mounted. In the absence of herbarium specimens, this illustration is the only original material and is designated as the lectotype for *C.reuteri*. According to Greuter (2008), C.leucophaeasubsp.reuteri (Rchb.f.) Dostál is an accepted name and applies to plants endemic to France and Italy. However, based on the lectotype, we agree with Tison and de Foucault (2014) in considering *C.reuteri* as a heterotypic synonym of C.leucophaeasubsp.leucophaea, a taxon endemic to southern France and northern Italy.

**28. *Centaureasubalbida* Jord. in Mém. Acad. Roy. Sci. Lyon, Sect. Lett. 1: 320. 1851 ≡ Centaureapaniculatasubsp.leucophaeavar.subalbida (Jord.) Rouy in Rev. Bot. Syst. Geogr. Bot. 2: 149. 1904. Type: FRANCE. Provence-Alpes-Côte d’Azur: Lyon [plants cultivated from seeds collected in Les Vans, Auvergne-Rhône-Alpes], 22 July 1869, *A. Jordan s.n.* (neotype, designated here: LY [barcode LY0375186], photo!; isolectotype: LY [barcode LY0825495], photo!, https://explore.recolnat.org/occurrence/FE21B1BB9A494C92B5E33BD5446CEEEE)**

In the protologue, Jordan (1851) provided a detailed description and reported Les Vans and Banne (Auvergne-Rhône-Alpes) as localities of occurrence. We searched in Jordan’s herbarium but, as for *C.mierghii*, we located just specimens collected from Jordan’s garden after the publication of the name. In the absence of original material, a neotype can be designated. The specimen LY0375186, in Jordan’s herbarium, was grown from seeds mature plants collected at Les Vans, one of the two localities mentioned in the protologue. This specimen shows pubescent leaves composed of several linear-oblong segments. The involucres are oblong-ovoid and the involucral bracts show yellow to brown appendages, with lateral upper cilia that exceed in length the central mucro. Since this morphology agrees with the protologue, the specimen LY0375186 is designated as the neotype for *C.subalbida*. Based on the neotype morphology, we agree with Greuter (2008) and Tison and de Foucault (2014) in considering *C.subalbida* as a heterotypic synonym of *C.stoebe*, a species widespread in central-eastern Europe.

## ﻿Additional information

### Author ORCIDs

Antonio Giacò https://orcid.org/0000-0001-8263-6018

Lorenzo Peruzzi https://orcid.org/0000-0001-9008-273X

### Data availability

All of the data that support the findings of this study are available in the main text.
